# Glibenclamide Enhances the Therapeutic Benefits of Early Hypothermia after Severe Stroke in Rats

**DOI:** 10.14336/AD.2017.0927

**Published:** 2018-08-01

**Authors:** Shuzhen Zhu, Xiaoya Gao, Kaibin Huang, Yong Gu, Yafang Hu, Yongming Wu, Zhong Ji, Qing Wang, Suyue Pan

**Affiliations:** ^1^Department of Neurology, Nanfang Hospital, Southern Medical University, Guangzhou, Guangdong, China; ^2^Department of Neurology, Zhujiang Hospital, Southern Medical University, Guangzhou, Guangdong, China

**Keywords:** stroke, cerebral edema, glyburide, glibenclamide, therapeutic hypothermia

## Abstract

Glibenclamide (GBC) is an antidiabetic drug that is in a class of medications known as sulfonylureas, which play critical roles in attenuating brain edema and reducing mortality in ischemic stroke patients. Therapeutic hypothermia (TH) is another robust neuroprotectant that prevents brain swelling and improves the neurological outcomes of stroke patients. However, whether the combination of GBC and TH can be used as a reliable neuroprotectant in ischemic stroke remains largely unknown. We used the middle cerebral artery occlusion (MCAO) rat model as well as oxygen and glucose deprivation-reoxygenation (OGD/R) endothelial cells as ischemic stroke models to investigate the efficacy and mechanisms of treating ischemic stroke with the combination of GBC and TH. The serum glucose, mortality rate, neurobehavioral functions, tight junctions, endothelial cells and inflammatory cytokines were evaluated in the stroke models after treatment with GBC, TH or the combination of them. After 5-hour occlusion and subsequent reperfusion, rats exhibited a large volume of hemispheric swelling and a high mortality rate. Stroke rats treated with the combined therapy did not exhibit hypoglycemia. The combination of GBC and TH exhibited synergistic neuroprotective effects in stroke rats that were associated with greater reductions in edema volume, better improvement in neurobehavioral functions, prevention of tight junction loss, and reduction of expression of the inflammatory cytokines COX-2 and iNOS. In OGD/R endothelia cells, the combination reduced endothelial cell death. This study demonstrated that both GBC and TH are neuroprotective after the severe stroke; however, combined therapy with GBC and TH enhanced the efficiency and efficacy of the effects of TH and GBC in the treatment of ischemia. This combined therapy may facilitate the clinical translation of TH management for severe stroke. The combination of GBC and TH seems to be a feasible and promising clinical strategy to alleviate cerebral injury following severe stroke.

Therapeutic hypothermia (TH) is one of the most robust neuroprotectants in ischemic stroke and has been proved to reduce infarct size, protect the blood brain barrier (BBB) from breakdown and alleviate edema formation in animal models [1]. The potential mechanisms may include the inhibition of post-ischemic hyperperfusion [2], attenuation of brain inflammation [3] and reactive oxygen species (ROS) production [4, 5], stabilization of beta-catenin [6], and reductions of both glutamate and glycine release [7]. However, the efficacy of hypothermia is insufficient. Su et al. demonstrated that mild hypothermia may improve neurobiological outcome in survivors but did not reduce mortality in severe stroke patients [8]. Another study found that TH had no additional benefit on functional outcomes in patients with space-occupying cerebral ischemia [9]. The limited efficacy of TH might be partially due to the relatively short therapeutic time window [4] and brief period of TH used [1]. Based on previous findings, we propose that early-onset, appropriately prolonged TH, and suitable medical treatment could likely enhance the efficacy of TH [10] if the TH neuroprotection is optimized.

Glibenclamide (GBC) is a sulfonylurea drug that is widely used for the treatment of type 2 diabetes mellitus (DM). Recently, some studies have revealed that this compound can be used to treat various diseases other than DM, including spinal cord injury [11], kidney ischemia[12], traumatic brain injury[13, 14], status epilepticus [15] due to its actions of regulating intracellular ROS, inflammation, and oncotic necrosis in neural cells. Currently, the role of GBC in treating malignant edema and stroke has received attention [16, 17], whereas the mechanisms are not fully elucidated. Additionally, our previous study demonstrated that GBC enhances the efficacy of delayed TH [18]; however, whether the combination of GBC with early TH provides additional neuroprotection compared to its use alone and its potential neuropathogenesis are not clear.

Therefore, in the present study, we sought to determine the following: 1) whether GBC enhanced the efficacy of early TH in terms of infarct volume, cerebral edema reduction, tight junction preservation and neurological function restoration in a severe ischemic stroke model; 2) how the inhibitions of inflammatory responses and cleaved Caspase 3 mediate the additional protection of combined treatment; and 3) whether the combination of GBC and TH provided more protection for endothelial cells than either treatment alone in an *in vitro* study.

## MATERIALS AND METHODS

### Animal Preparation and MCAO Model

Male Sprague-Dawley rats weighing between 280 and 320 g were used in this study (Southern Medical University, Guangzhou, Guangdong). Before surgery, rats were trained with spontaneous locomotor activities and locomotor tests daily for 3 days. Then, rats underwent middle cerebral artery occlusion (MCAO) as we have described previously [19]. Animals were anesthetized with 5% isoflurane in 70% N_2_ and 30% O_2_ and maintained on 1.5% isoflurane anesthesia using a facemask. After anesthesia, the rats were placed on a temperature feedback heating pad (setting temperature was 38.0 °C, RWD Life Science Co., Shenzhen, China), which automatically regulate the rectal temperature to 38.0 °C only and prevent hypothermia or overheating. The brain temperature was monitored using a cerebral thermometer probe (THERMOMETER, BAT7001H, Physitemp Instruments, Inc. Clifton, New Jersey, United States) that was inserted through a burr hole (3-mm posterior and 3-mm lateral to bregma and 4 mm below the skull surface) as we have described previously [20]. Bilateral burr holes (1 mm in diameter) were drilled 6 mm lateral and 1 mm posterior to bregma, and a laser Doppler flowmeter (Moor Instruments Ltd., Devon, UK) probe was positioned above the surface of the hemisphere to monitor the cerebral blood flow (CBF) [19]. Venous blood from the tails was collected to measure blood glucose with a commercially available glucose monitor (Freestyle Lite; Abbott, Abbott Park, IL). Next, a silicone-coated suture (MCAO sutures, Jialing Corp., Guangzhou, China) was gently inserted through the external carotid artery to the internal carotid artery to occlude the MCA for 5 hours. The success of occlusion was determined by a decrease in blood flow to less than 30% of the baseline value. In the sham-operated rats, a suture was inserted to the opening of the middle cerebral artery and then immediately withdrawn.

As illustrated in Fig. 4A, at 30 minutes after MCAO, rats were randomized to the vehicle, GBC, TH or combined groups (GBC+TH). GBC (Sigma, St. Louis, MO) was dissolved in 0.01% dimethyl sulfoxide (DMSO) saline solution and intraperitoneally administered with a loading dose of 10 μg/kg at 295 minutes after MCAO onset and once daily thereafter. Rats in the vehicle group received an equivalent volume of DMSO saline solution. Rats in the TH-treated group were placed on an ice pad at 30 minutes after MCAO onset for the first 30 min and on a temperature control pad (approximately 34°C) (RWD Life Science, Shenzhen, China) for another 360 min and then gradually rewarmed from approximately 34 °C to 37 °C at a speed of approximately 1 °C/hour. The sample sizes of each group are provided in Fig. 4B.

### Neurological outcome, edema, and infarct volume evaluations

On the third day after stroke, the mortality rate was calculated with the following formula: 3-day mortality rate = the total number of dead rats in 3 days/the total number of rats enrolled on the first day. The surviving rats were used for motor function tests. Spontaneous locomotor activity was tested with an open field activity monitoring system according to the description by Liu [21, 22]. Motor coordination was tested with an accelerating rotarod as described in Atif’s study [21], which was started at 2 rpm and linearly accelerated to 20 rpm within 300 seconds. The total distance traveled and the latency to fall off the rotarod were determined as percentages of the pre-surgery levels. At 24 hours after MCAO onset, a series of 2-mm-thick coronal sections were obtained and stained with 1% 2,3,5-Triphenyltetrazolium chloride (TTC) (Sigma), and the swelling and infarction volume were analyzed according to our previous description[18].

**Table 1:** Effects of hypothermia and gbc on cerebral blood flow, blood glucose, brain and rectal temperature.

Parameters	Time points	Sham	Vehicle	GBC	TH	GBC+TH
CBF reduction (%)	Baseline (-15 min)	100.0 ± 4.3	100.0 ± 4.1	100.0 ±16.9	100.0 ±11.5	100.0± 23.5
	0 min	98.9 ± 7.4	20.6 ± 6.8*	19.1 ± 7.5	21.7 ± 4.7	20.2 ± 4.7
	45 min	99.2 ± 5.8	16.4 ± 2..5*	16.8 ± 2.0	17.1 ± 1.3	16.4 ± 4.0
	315 min	100.0± 5.3	86.0 ± 4.4*	91.8 ± 3.0	104.2 ± 9.8	95.1 ± 19.1
Glucose (mg/dl)	Baseline (-30 min)	143.7 ± 5.7	145.5 ± 8.1	138.3± 13.7	147.9± 20.6	137.72 ± 19.1
	0 min	144.6 ± 8.3	165.9±14.9*	161.4 ± 7.0*	160.2± 47.5*	161.7± 37.87*
	60 min	136.5 ± 10.6	137.6 ± 23.8	138.3± 14.2	134.4± 18.4	142.5 ± 28.4
	300 min	139.8 ±11.8	141.3 ± 34.4	149.4 ±12.5	160.8± 27.6*#	174.6± 30.0*#
	480 min	141.6 ± 9.2	141.4 ± 22.2	130.2 ± 5.9	171.0± 38.0*#	196.0 ± 36.4*#
Brain temperature (°C)	Baseline (-30 min)	33.1 ± 0.5	33.1 ± 0.2	33.1 ± 0.2	33.3 ± 0.3	34.0 ± 0.5
	0 min	33.2 ± 0.2	33.2 ± 0.2	33.5 ± 0.3	33.2 ± 0.4	33.6 ± 0.5
	60 min	34.9 ± 0.3	35.2 ± 0.2	35.1 ± 0.2	31.6 ± 0.2*#	31.9 ± 0.5*#
	315 min	36.5 ± 0.4	36.8 ± 0.4	36.2 ± 0.5	32.3 ± 0.3*#	32.6 ± 0.3*#
Rectal temperature (°C)	Baseline (-30 min)	36.7 ± 0.3	36.8 ± 0.2	36.8 ± 0.2	36.8 ± 0.2	37.0 ± 0.2
	0 min	37.0 ± 0.3	37.0 ± 0.4	37.0 ± 0.5	36.7 ± 0.2	37.2 ± 0.3
	60 min	37.5 ± 0.5	37.7 ± 0.7	37.5 ± 0.2	34.5 ± 0.3*#	34.0 ± 0.3*#
	315 min	38.3 ± 0.2	39.0 ± 0.4	39.2 ± 0.3	35.3 ± 0.3*#	35.3 ± 0.3*#

The values are expressed as mean ± SD. Statistical significance was determined by a one-way ANOVA with Bonferroni correction.

* *P* < 0.05 versus sham group.

# *P* < 0.05 versus vehicle group using between group comparisons with Bonferroni correction. CBF, cerebral blood flow; GBC, glibenclamide; TH, therapeutic hypothermia.

### Endothelial cell culture and OGD/R

Cells of the immortalized mouse brain endothelial cell line bEnd.3 were cultured in Dulbecco’s modified Eagle’s medium (DMEM) (Gibco™, Thermo Fisher Scientific) supplemented with 10% fetal bovine serum (Gibco™). Oxygen glucose deprivation and reperfusion (OGD/R) was performed after 3 days of culture according to our previous description [23]. After 5 hours, the cells were reperfused by placing them back into a normoxic incubator at 37 °C or 34 °C for 3, 4.5 or 12 hours with vehicle or GBC culture medium. Subsequently, the cells in the 34 °C incubator were returned to a normothermic incubator for an additional 16, 14.5 or 7.0 hours [24].

**Figure 1.**
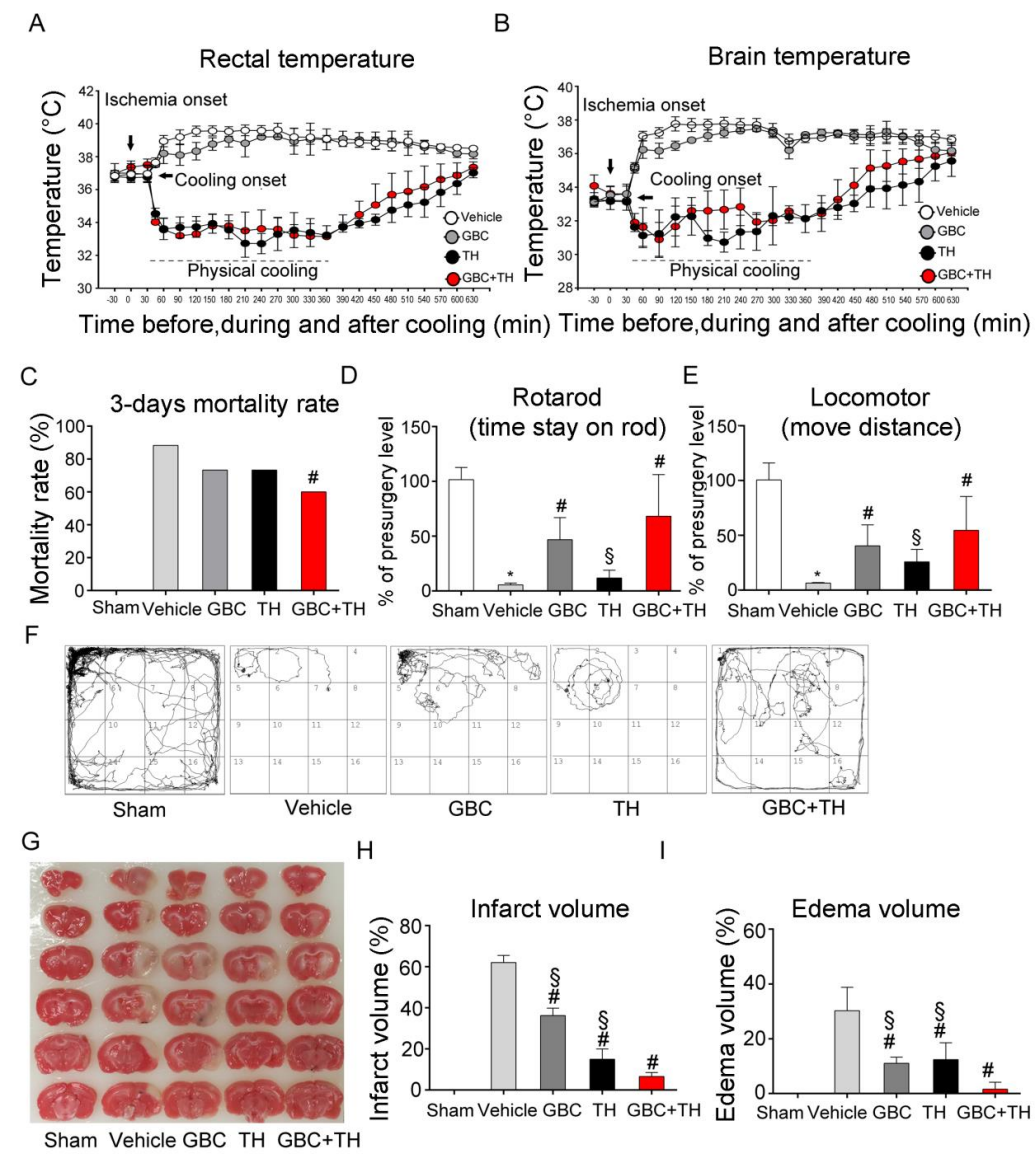
Effects of the combination of GBC and TH on neurological outcomes, edema volume (**A & B**) Rectal and brain temperatures were monitored in the MCAO rats that were treated with vehicle, GBC, TH or GBC+TH. n= 9. (**C** - **F**) Mortality rate and motor function were examined on day 3 post-MCAO. n=7-9. (**G** - **I**) Infarction and edema volume were evaluated on day 1 post-MCAO. The values are expressed as the means ± the SDs. ^*^P< 0.05 compared to sham; *^#^P< 0.05* compared to vehicle; and *^§^P< 0.05* compared to GBC+TH. GBC, glibenclamide; TH, therapeutic hypothermia; MCAO,middle cerebral artery occlusion.

### Cell viability assay, cell morphology, and death detection

Twenty-four hours after OGD, the cell viability was tested with a CCK-8 kit (Dojindo Laboratories, Tokyo, Japan) according to the manufacturer’s instructions. Viability is expressed as a percentage relative to the control cells. Cell morphology was observed with an inverted microscope (Olympus, Japan), and cell death was detected with propidium iodide (PI; Sigma, USA) staining with the method of Atif [21].

### Western blot

Eight hours after MCAO onset, protein was collected from the ipsilateral hemispheric cortex and immuno-probed with primary antibody (Supplementary Data Table 1) and horseradish peroxidase (HRP)-conjugated secondary antibody. The protein bands were visualized on an ECL^®^ hyper film (GE Healthcare, Little Chalfont, UK) by chemiluminescence. The images were recorded using an Automated Kodak In-Vivo Imaging system (Carestream Health, New Haven, USA).

### Statistical analysis

The statistical analyses were performed with independent t-tests or one-way ANOVAs followed by post hoc tests using the Bonferroni correction with SPSS version 13 (IBM, Armonk, NY). The data are represented as the means ± the standard deviations (SDs). The mortality analysis was performed with the chi-square test. The graphs were made using GraphPad Prism 6 software (GraphPad Software, San Diego, CA). *P*-values <0.05 were considered significant.

**Figure 2.**
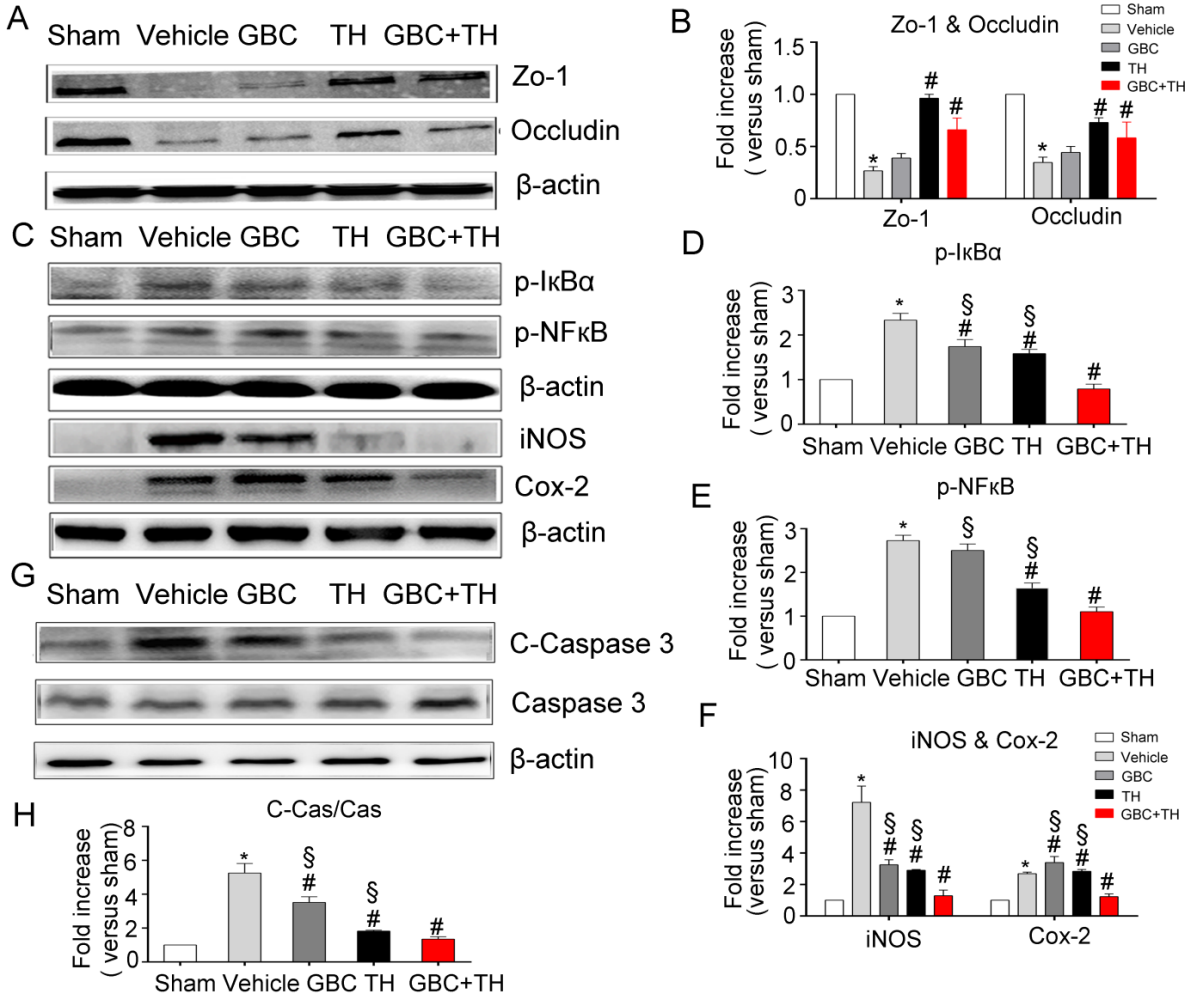
Effects of the combined treatment with GBC and TH on the tight junction proteins, inflammatory response and cleaved-Caspase 3 The effects of GBC and TH on the expressions of Zo-1, Occludin, p-IºBα, p-NF-ºB P65, iNOS, Cox-2, and Cleaved Caspase 3 in the rat brains 8 hours post-injury. (**A, C, G**) Representative protein bands and densitometry data (**B, D, E, F, H**) from Western blots. β-actin was used as the loading control. n=5. The values are expressed as the means ± the SDs. *P<0.05 compared with sham; *^#^P<0.05* compared with vehicle; and *^§^P<0.05* compared to GBC+TH. GBC, glibenclamide; TH, therapeutic hypothermia; ZO-1, Zonula occludens-1.

## RESULTS

### CBF, blood glucose, and temperature monitoring

As presented in Table 1, the baseline cerebral blood flow (CBF), blood glucose and temperature were comparable among the MCAO groups. The CBF was reduced to a level below 20% of the normal state after occlusion, and an increase of 97% was observed after reperfusion, indicating an incomplete reperfusion. The blood glucose increased after ischemia and almost reached the baseline levels 60 min later. Notably, no hypoglycemia was detected in the GBC-treated rats. As expected, post-stroke hyperthermia (rectal temperature 38-39 °C, brain temperature 36-37 °C) occurred in MCAO rats (Fig. 1 A and B) [10], and TH prevented pyrexia and decreased the rectal temperature to 33.6 ± 0.7 °C and the brain temperature to 31.1 ± 0.7 °C within 30 minutes.

### Neurological outcomes, edema, and effects on tight junctions

At 3 days after MCAO, 53 of 60 rats had died in the vehicle group (88.3% mortality) vs. 18 of 30 rats in the GBC+TH group (60% mortality) (*Pearson chi-square=9.79, P=0.02;* Fig. 1 C). Among the surviving rats, the GBC+TH-treated rats stayed on the rod longer and travelled a greater distance than those in the vehicle group (*P < 0.05;* Fig. 1 D–F). These results suggest that the GBC+TH treatment decreased the mortality rate and improved motor function at 3 days after stroke.

**Figure 3.**
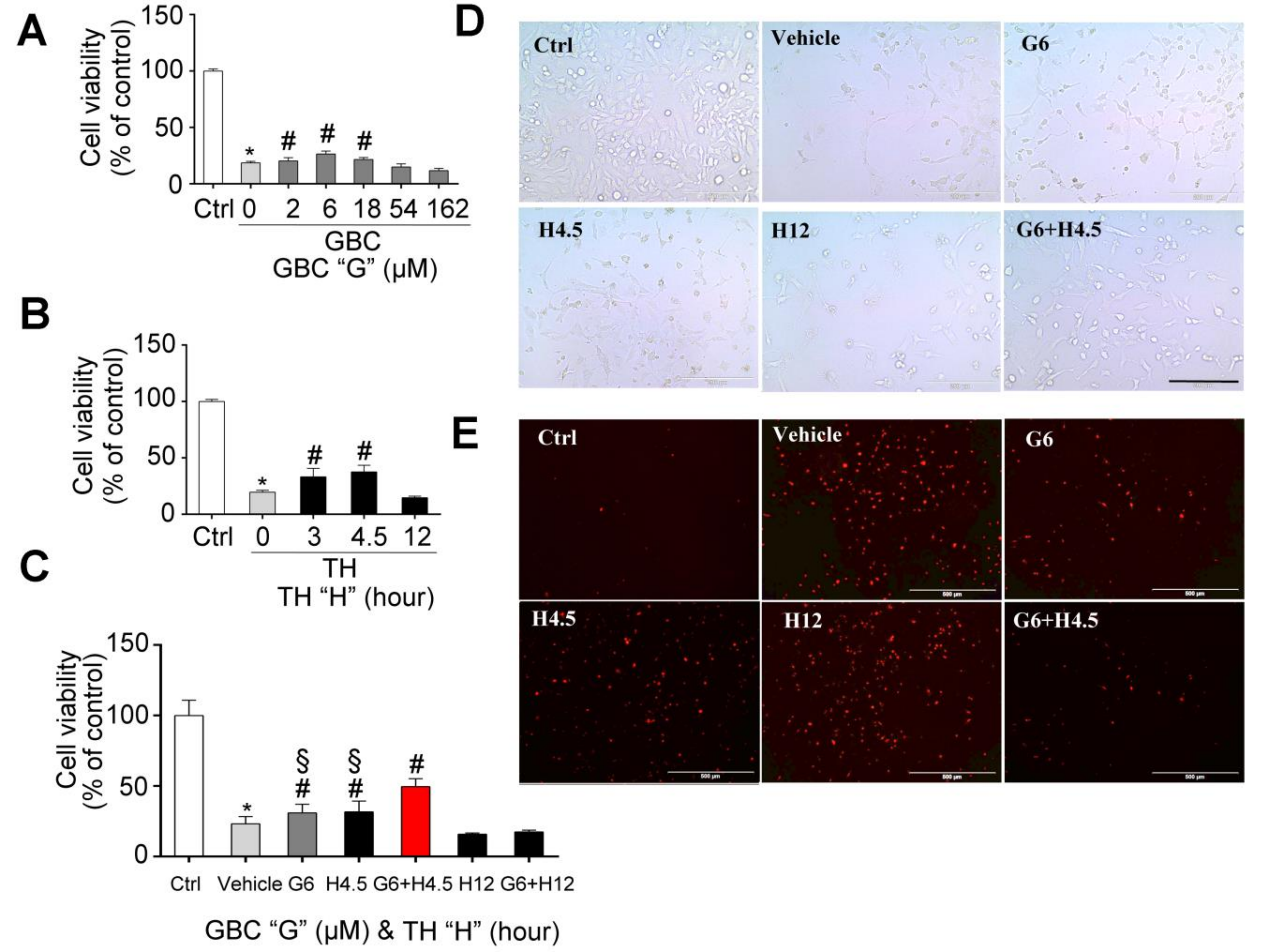
The effects of the combination of GBC and TH on the stabilization of endothelial cells (**A-C**) Various concentration of GBC (**A**), different durations of therapeutic hypothermia (**B**) and their combinations (**C**) on the on the viability of endothelial cells 24 hours post-OGD. (***D-E***) Representative cell morphology observed with an inverted microscope (**D**) and representative cell morphology of cell death by PI staining 24 hours post-OGD (**E**). The values are expressed as the means ± the SDs of three experiments. ^*^P < 0.05 compared to sham; *^#^P < 0.05* compared to vehicle; and *^§^P< 0.05* compared to GBC+TH. OGD, oxygen glucose deprivation; GBC, glibenclamide; TH, therapeutic hypothermia; Ctrl, control; PI, propidium iodide.

One day after stroke, large infarcts formed in the vehicle-treated rats (≈60% brain damage in the ipsilateral hemisphere). The GBC and TH treatments each significantly reduced the infarct volume (Fig. 1 G and H) (*P<0.05*). The results presented in Fig. 1 G and I also revealed a large swelling (30.2%±8.6%) in the vehicle-treated group; however, treatment with GBC+TH (1.6%±2.6%) produced a significant reduction in swelling (*P<0.001*) compared with the TH (12.4%±6.2%) and GBC (11%±2.3%) alone groups. After stroke, tight junction proteins were lost, which may have caused a disruption of the blood-brain barrier (BBB) and secondary vasogenic edema. Both TH and the combination treatment preserved the tight junction proteins (*P<0.05;* Fig. 2 A and B).

### Inflammatory cytokines and cleaved Caspase 3

Shortly after ischemic stroke onset, inflammatory cytokines accumulate in the brain and may lead to endothelial apoptosis and death. The results in Fig. 2 C–H demonstrated that GBC+TH elicited greater suppressions of the upregulations of p-NF-κB, p-IκBα, COX-2, iNOS and cleaved-Caspase 3. These results indicate that inflammatory response inhibition might be associated with the synergistically protective effect of GBC and TH on endothelial cells after ischemic stroke.

### Effects of the combined treatment on endothelial cell death

Endothelial cells are important components that maintain the integrity of BBB. The results in Fig. 3 A–E demonstrate that the death of endothelial cell was attenuated following treatment with the combination of GBC (6 μM) and TH (4.5 hours duration, target temperature at 34 °C) compared to monotherapy treatment. These results might partly explain why the combined therapy with GBC and TH produced better effects on edema reduction compared with the monotherapy.

## DISCUSSION

In this study, we investigated the effects of GBC and TH in *in vivo* and *in vitro* ischemic models and obtained four principal findings: 1) the combination of low-dose of GBC and TH did not lead to the hypoglycemia; 2) in a severe ischemic stroke rat model, we noted that the combination of GBC and TH improved the functional recovery; 3) the combination significantly attenuated the cerebral edema and protected the tight junction proteins; and 4) the combination of GBC and TH provided better protection for endothelial cells and attenuated the expression of inflammatory factors as well as cleaved Caspase 3.

Although several lines of evidence demonstrate that GBC and TH may provide neuroprotection against cerebral infarction by affecting various mechanisms of neuropathogenesis [25, 26], the efficacy of single treatment still needs to be explored with large clinical trials. Whether the combination of GBC and TH could provide greater neuroprotective effects in ischemic subjects needs to be further explored. Our previous study demonstrated that, in a mild ischemic stroke model, i.e., a 3-hour MCAO occlusion in rats, delayed TH (6 hours post-stroke onset) alone failed to decrease cerebral edema and infarct volume, whereas the combination of TH and GBC improved neurological outcomes and ameliorated BBB disruption. Interestingly, the severe ischemic stroke model, i.e., a 5-hour MCAO model, produced large infarcts that more closely resemble malignant and fatal human infarction than the average sized stroke. In this study, in order to enhance the efficiency of hypothermia, we initiate the TH at 0.5 hour post-stroke. The neuroprotective duration of TH will last more than 5 hours [27]. GBC administration at 5-hour occlusion may enhance the effects of TH. This finding is clinically meaningful because it strongly implies that early onset TH may correlate with improved outcomes and mortality in patients with severe stroke, and TH could be clinically used to treat patients with severe strokes. More importantly, we demonstrated that TH, in combination with GBC, led to a more effective amelioration of the ischemic injury, which included improving the functional recovery and neurobehavioral function, reducing the mortality rate and reducing the edema volume, than either therapy given alone. This combination produced enhanced neuroprotective effects in ischemic rats. These data support the strategy of combining TH with pharmacological GBC to increase the clinical feasibility, efficacy, and safety of the treatment of patients with stroke. Considering that mild hypothermia decreases the total clearance of glibenclamide after the administration of low-dose glibenclamide in normal rats [28], the effects of the combination of GBC with TH on the plasma glucose level needs to be examined. This study first evaluated the safety properties of the combined treatment with GBC and TH and confirmed that this combination did not enhance the risk of hypoglycemia in the severe stroke rat model.

To explore whether the synergistic effects of GBC and TH on edema reduction were associated with BBB protection, tight junction proteins and endothelial cells, which are two of the most important components of the BBB, were evaluated. Impressively, we noted that both GBC and TH prevented the deaths of endothelial cells and decreased the loss of tight junctions. The combination exerted no additional benefit on tight junction’s expression; however, whether the combined therapy delivered more protection in alleviating the translocation of tight junction proteins needs to be further explored. This finding strongly suggests that the synergistic effects of ischemic neuroprotection may at least partially result from endothelial cell stabilization.

**Figure 4.**
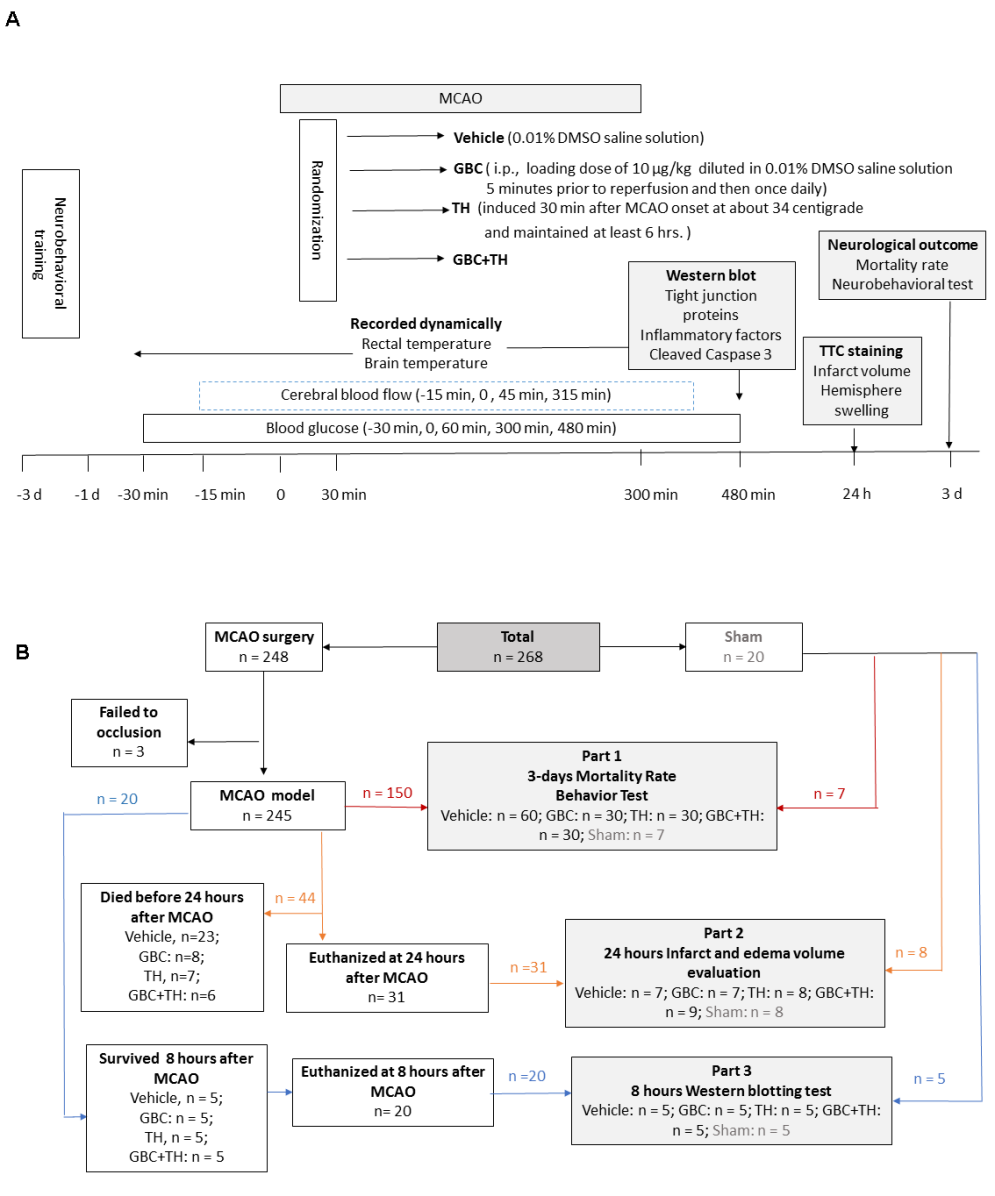
Experimental procedure and measurements at baseline and during MCAO and reperfusion **A**), Cerebral blood flow and blood glucose were intermediately detected, and rectal and brain temperatures were continuously monitored. **B**), Flow diagram of the experimental groups. DMSO = dimethyl sulfoxide, GBC = glibenclamide, i.p. = intraperitoneal injection, TH = therapeutic hypothermia, MCAO = middle cerebral artery occlusion, CBF = cerebral blood flow, TTC = 2, 3, 5-Triphenyltetrazolium chloride.

The upregulation of inflammation factors, such as iNOS, Cox-2, in acute stage of cerebral injury was detrimental to the pathophysiology of cerebral injury [26, 29-32]. Both TH and GBC have been reported to exert anti-inflammatory and anti-apoptosis effects in cerebral injury [33-37]. In our study, we noted significant decreases in the p-IκBα, pNFκB, iNOS and Cox-2 levels in the MCAO rats with TH treatment and a relatively slight decrease after low dose GBC treatment compared with the MCAO rats, suggesting that early TH treatment provides an anti-inflammatory response. In our study, we used a relative low dose of GBC (10 μg/kg.d), which may partially be responsible for the weak effect of GBC in anti-inflammation. More interestingly, the combination of GBC and TH led to more pronounced decreases in the levels of p-IκBα, pNFκB, iNOS and Cox-2 when compared with GBC or TH alone, which indicates greater efficacies of the anti-inflammatory and anti-apoptosis responses following the administration of this combination compared with either treatment used alone. Additionally, a remarkable decrease in cleaved Caspase-3 was observed in the group that received the combination of GBC and TH compared with the groups that received TH or GBC alone, which indicates that this combined treatment may provide a greater anti-apoptosis effect than either TH or GBC alone.

## Summary

For the first time, this study demonstrated the beneficial effects of a combination therapy of TH and GBC for treating stroke. The combination of TH and GBC was associated with greater edema reduction, prevention of cellular and brain damage, improvements in functional recovery, synergistic neuroprotection of endothelial cells and suppression of the inflammatory response in MCAO ischemic rats. The combined treatment could represent a promising approach to creating more effective and safer hypothermia therapies for patients with brain injuries.
